# Responses of Coagulant Type, Dosage and Process Conditions to Phosphate Removal Efficiency from Anaerobic Sludge

**DOI:** 10.3390/ijerph19031693

**Published:** 2022-02-01

**Authors:** Dae Wook Kim, Sung Il Yu, Kyuyong Im, Juhee Shin, Seung Gu Shin

**Affiliations:** Department of Energy Engineering, Future Convergence Technology Research Institute, Gyeongsang National University, Jinju-daero, Jinju 501, Korea; wd6780@gmail.com (D.W.K.); tjd3427@naver.com (S.I.Y.); jayoou03098@naver.com (K.I.); shinjh@gnu.ac.kr (J.S.)

**Keywords:** struvite, coagulation, response surface methodology, central composite design, optimization

## Abstract

Phosphorus, a crucial component of life, may cause eutrophication if it is discharged untreated into the aquatic ecosystem. Phosphate (PO_4_^3-^) may exist at an elevated level in anaerobic digestion (AD) effluents and can lead to the clogging of pipes by forming struvite crystals. This study was conducted to assess the responses of coagulant type, dosage and process conditions to phosphate removal efficiency from anaerobic sludge. The experiments were performed in two steps. First, a sensitivity test was conducted to compare five coagulant types (alum, poly-aluminum chloride (PAC), FeCl_2_, FeCl_3_ and PAC + FeCl_3_) at standard coagulation conditions. The results showed that PAC would be the best coagulant among the tested, while a combination of PAC and FeCl_3_ may be beneficial under circumstances. Second, an optimization study was performed for PAC using response surface methodology employing central composite design. Among the three independent variables (coagulant dosage, slow mixing duration and agitation speed), the dosage was the sole significant variable for phosphate removal efficiency, while the other two had limited effects. A future study to optimize the rapid mixing conditions would give additional insights into the process. The results of this study may be useful to design a process to counteract phosphate discharges from AD plants, as well as to reduce the risks of pipe clogging and maintenance problems due to crystalline struvite formation in the later stage of AD.

## 1. Introduction

As a component of nucleic acids and many other biomolecules, phosphorus (P) is an essential element for living organisms [1]. Thus, phosphorus is regarded as one of the macro-nutrients that are required for the growth of microorganisms in biological waste and wastewater treatment processes, such as anaerobic digestion (AD) [2]. Conversely, an excessive level of phosphorus in the water bodies, rooted from point and non-point sources, may lead to eutrophication and serious algal blooms in the aquatic environment [3]. To prevent this, phosphorus level in discharges is strictly regulated in most countries.

AD involves strict and facultative anaerobic microbes to degrade organic materials under reducing environments. Although AD can successfully convert organic carbon into biogas, a combustible, methane-rich fuel, the biochemical pathways of AD do not show significant effects on phosphorus removal [4]. Instead, organic phosphorus in the feedstock is largely decomposed into a more available form, such as phosphate (PO_4_^3-^), and remains in the digestate. Therefore, a direct discharge of the digestate has the potential to raise aforementioned environmental issues to the aquatic ecosystem.

Struvite is a phosphate mineral with the formula of NH_4_MgPO_4_·6H_2_O [5]. Crystalline struvite is often formed in AD plants and causes problems such as pipe clogging [6]. Once clogged, the pipes need extensive maintenance efforts to remove struvite crystals. Thus, preventing its formation has been considered as a precautionary alternative [7]. Removing one of the precursors (i.e., ammonium, magnesium and phosphate) from anaerobic sludge can limit struvite precipitation in the plants [8].

Coagulation-flocculation is a widely practiced treatment method to remove solids and some ionic species from wastewater discharges [9]. Coagulation of phosphate in the anaerobic sludge can be accomplished by establishing a coagulation process after the digestion stage. The effective coagulants may include Al-based ones, such as alum and poly-aluminum chloride (PAC), and Fe-based ones, such as FeCl_2_ and FeCl_3_ [10]. While the former (Al-based) is known to have excellent cohesiveness, the latter (Fe-based) has a wide range of usable pH and is less affected by low temperature. In addition, the dosage of coagulant, the agitation strength and the reaction time should affect the phosphate removal efficiency. Selection of optimal coagulant and process conditions can lead to an efficient phosphate removal from anaerobic effluents and control of the formation of struvite [11].

This study was designed to collect information to establish the coagulation process for phosphate removal from AD sludge. The experiments were conducted in two stages. First, four different compounds (alum, PAC, FeCl_2_ and FeCl_3_) and one combination of them (PAC with FeCl_3_) were compared as coagulant. Second, two best-performing coagulants (PAC and FeCl_3_) were further evaluated for optimization by their working conditions: dosage, agitation speed and reaction time. Response surface methodology (RSM) using a central composite design (CCD) was employed for the optimization [12].

## 2. Materials and Methods

### 2.1. Sludge and Coagulants

Anaerobic sludge was collected from a domestic wastewater treatment plant (Jinju, South Korea). The characteristics of the sludge, including the total phosphorus and phosphate-P (PO_4_^3-^-P) concentrations, are shown in Table 1. The coagulants used in this study were alum (Al_2_(SO_4_)_3_·18H_2_O), PAC (Al_2_(OH)_5_·Cl), FeCl_2_ (FeCl_2_·4H_2_O) and FeCl_3_. The coagulants were prepared as stock solutions (300 g/L; 170 g/L for PAC) before use.

### 2.2. Coagulant Sensitivity Experiment

The sensitivity of coagulation according to coagulant type was tested using a jar-tester (C-JT-1, Changshin Science, South Korea). Anaerobic sludge (300 mL) was put in a beaker (1 L) and kept at 30 °C during the reaction. Five doses (1, 2, 3 and 5 molar ratios of either Al or Fe to phosphate) were tested using alum, PAC, FeCl_2_ and FeCl_3_ as the individual coagulant. In addition, a mixture of PAC and FeCl_3_ (PAC + FeCl_3_) was also tested; in this case, a dose of *n* ratio meant that both Al and Fe were added at *n* molar ratio to phosphate, summing up to *2n* total metal ions. The total reaction time was set as 18 h, where 10 min of initial rapid mixing was followed by 17 h 50 min of slow mixing. The agitation speed for the rapid mixing was fixed at 150 rpm, while two different agitation speeds (20 and 50 rpm) were tested for the slow mixing period. The pH was checked after the coagulant was added and the rapid mixing (10 min) was completed; if the pH was below 7.0, it was corrected to 7.0 by adding NaOH (5 M). After completion of the reaction, the remaining phosphate concentration was measured by spectrophotometry using HS-PO_4_(P)-H and HS-PO_4_(P)-L kits (Humas, South Korea).

The remaining phosphate concentration profiles according to the dosage were evaluated using an exponential decay model (Equation (1)):

*y* = *y*_0_ + *a* exp(−*bx*) (1)

where *y* is the phosphate concentration, *y*_0_ is the persisting phosphate concentration, *a* is the removable phosphate concentration, *b* is the decay constant and *x* is the dose.

In addition, potential interaction of the two coagulants in the combination trial (PAC + FeCl_3_) was assessed by comparing the phosphate removal efficiencies of the three trials (PAC, FeCl_3_ and PAC + FeCl_3_). First-order (liner), 1.5th-order (interaction) and second-order (quadratic) models were developed to fit the results of the combination trial to the results of the mono-coagulant trials (Equations (2)–(4)):

*R_P+F_* = *β*_0_ +* β_P_R_P_* + *β_F_R_F_* (2)

*R_P+F_* = *β*_0_ + *β_P_R_P_* + *β_F_R_F_* + *β_PF_R_P_R_F_* (3)

*R_P+F_* = *β*_0_ + *β_P_R_P_* + *β_F_R_F_* + *β_PF_R_P_R_F_* + *β_PP_R_P_*^2^ + *β_FF_R_F_*^2^ (4)

where *R_P+F_* is the phosphate removal efficiency of the combination trial (PAC + FeCl_3_), *R_P_* and *R_F_* are, respectively, the phosphate removal efficiency of the PAC and FeCl_3_ trials and *β*’s are the coefficients. The modeling procedure was conducted using R software package (R Core Team, Austria).

### 2.3. Optimization Using RSM

Following the sensitivity experiment, PAC and FeCl_3_ were further studied for optimization using RSM. Jar tests were performed as Section 2.2 with the following modifications. Three independent variables were investigated for their responses to phosphate removal efficiency (Table 2 for PAC and Table S1 for FeCl_3_): coagulant dosage (“Dose”, *X*_1_), slow mixing duration (“Time”, *X*_2_) and agitation speed during the slow mixing (“RPM”, *X*_3_). The range of the variables was determined following the sensitivity test and some additional preliminary experiments (data not shown): 1 to 3 for *X*_1_, 20 to 180 min for *X*_2_ and 20 to 70 rpm for *X*_3_. A CCD was developed using three levels (−1, 0, +1) of each variable: Dose (1, 2, 3), Time (20, 100, 180 min) and RPM (20, 45, 70 rpm). The face-centered design contained 18 trials: eight factorial points, six axial points and four center points (quadruplicate).

A quadratic model was derived to describe the responses of the independent variables (Dose, Time and RPM) with the residual phosphate-P concentration ([PO_4_-P]) (Equation (5)):

(5) $$Y_{m}=\beta_{0}+\overset{n}{\underset{i=1}{\sum }}\beta_{i}X_{i}+\overset{n}{\underset{i=1}{\sum }}\beta_{ii}X_{i}^{2}+\overset{n-1}{\underset{i=1}{\sum }}\overset{n}{\underset{j=i+1}{\sum }}\beta_{ij}X_{i}X_{j}$$

where *Y_m_* is the response variable (i.e., [PO_4_-P]), and *X_i_* and *X_j_* are the independent variables. The *β*_0_, *β_i_*, *β_ii_* and *β_ij_* are, respectively, the constant coefficient, the linear coefficients, the quadratic coefficients and the interaction coefficients.

The statistical significance of the model was expressed as the coefficient of determination *R*^2^. To derive the interaction between the independent variables and the response variable, an analysis of variance (ANOVA) was conducted. Statistical significance was confirmed by the *F*-test and the model term was evaluated as a *p*-value with a 95% confidence level. In addition, the optimal point was searched and visualized by a contour plot. Minitab software (version 17) was used for experimental design and statistical analysis.

## 3. Results and Discussion

### 3.1. Sensitivity Test

**Figure 1:**
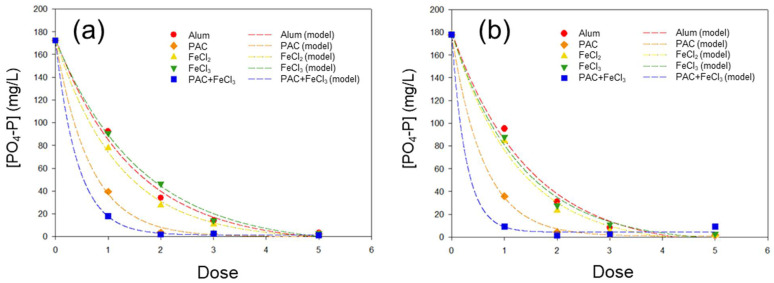
The residual [PO_4_-P] profiles and model predictions according to the coagulant type. Agitation speed of (**a**) 50 rpm or (**b**) 20 rpm was applied for the slow mixing (17 h 50 min) after the initial rapid mixing (150 rpm) of 10 min.

The five coagulant trials (alum, PAC, FeCl_2_, FeCl_3_ and PAC + FeCl_3_) were compared for their phosphate removal efficiency from the anaerobic sludge (Figure 1). Overall, the phosphate removal efficiency elevated as the coagulant dosage increased. This tendency was confirmed by the high coefficient of determination (*R*^2^ > 0.99) for the exponential decay models (Table 3). Total phosphorus was removed between 9% and 19% compared to the initial value (1197 mg/L).

The stoichiometric requirements for complete removal of phosphate (PO_4_^3−^) are 1.0 for Al^3+^ or Fe^3+^ and 1.5 for Fe^2+^. However, an elevated dose of 1.5–2.5 has been suggested as a practical guideline for efficient coagulation using Al^3+^ [13]. Similarly, removal efficiencies of ~90% or higher were observed at a coagulant dosage of 3 in this study (Figure 1), and nearly complete phosphate removal was achieved for all trials at a dosage of 5.

Among the individual coagulants (alum, PAC, FeCl_2_ and FeCl_3_), PAC showed the most efficient phosphate removal, according to coagulant dosage (Figure 1). The phosphate removal efficiencies for PAC were 77–80% or 98% at doses of 1 or 2, respectively, while the counterpart efficiencies were 47–55% (dose 1) or 73–87% (dose 2) for alum, FeCl_2_ and FeCl_3_. Likewise, the exponential decay constants (*b*; Equation (1)) of PAC was twice as high as those of the other chemicals at both 50 and 20 rpm agitation speeds (Table 3). The higher coagulation performance of PAC over the other chemicals may be attributed to its polymeric characteristics [14]. In addition, PAC has been gaining more attention as a coagulant in the water industry for its “bridging” ability and lesser sludge production [15].

The co-treatment using PAC and FeCl_3_ certainly improved the phosphate removal compared to the mono-treatments, with 90–95% efficiency for dose 1 and 99% for dose 2 (Figure 1). However, dose “1” of PAC + FeCl_3_ in this study means dose 1 of PAC plus dose 1 of FeCl_3_; therefore, compensation for the double dosage is necessary for comparison. The compensated exponential decay constants for PAC + FeCl_3_ were 1.819 at 50 rpm or 1.173 at 20 rpm, which were above (1.650; 50 rpm) or below (1.522; 20 rpm) the decay constants for PAC only (Table 3). A similar recipe, poly-aluminum ferric chloride (PAFC), has been reported as an efficient coagulant for organic and particulate matters in wastewater [16]. However, insufficient mixing at 20 rpm might have limited the coagulation performance of the combinatory compounds in this study.

**Figure 2:**
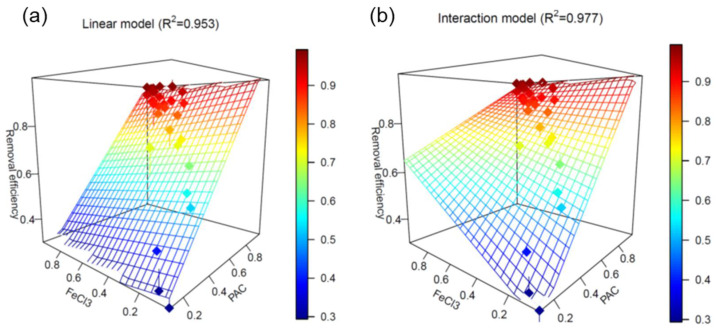
Three-dimensional plots of the (**a**) linear and (**b**) interaction models for the phosphate removal efficiency using coagulant combination (PAC + FeCl_3_) to individual coagulants (PAC or FeCl_3_).

To speculate if any interaction existed between PAC and FeCl_3_ on the coagulation of phosphate, three models (linear, interaction and quadratic) were compared to fit phosphate removal efficiency data between the co- and mono-treatments. Among the three models, the interaction model showed the best fitting (*R*^2^ = 0.977), followed by the linear (*R*^2^ = 0.953) and the quadratic (*R*^2^ < 0.8; data not shown) (Figure 2). The interaction model had the following form (Equation (6)):

*R_P+F_* = 0.1349 + 0.9361*R_P_* + 0.4824*R_F_* − 0.5808*R_P_R_F_* (6)

where *R_P+F_* is the phosphate removal efficiency of the combination trial (PAC + FeCl_3_), and *R_P_* and *R_F_* are the phosphate removal efficiency of the PAC and FeCl_3_ trials, respectively. These results imply that PAC (coefficient of 0.9361) was about twice more influential than FeCl_3_ (coefficient of 0.4824) to the overall phosphate removal efficiency when combined. Interestingly, the coefficient for the interaction term was negative (−0.5808), possibly due to the negative apparent interaction of the two coagulants (1.173 < 1.522) for the 20 rpm trials (Table 3).

### 3.2. Responses of Dosage, Reaction Time and Agitation Speed

The sensitivity experiment showed that PAC and FeCl_3_ were the best coagulant, in terms of the dosage-to-removal efficiency, to precipitate phosphate from anaerobic sludge. To explore the optimum conditions for phosphate coagulation, an RSM with face-centered CCD was employed. Three independent variables were selected for investigation: coagulant dosage (“Dose”, *X*_1_), slow mixing duration (“Time”, *X*_2_) and agitation speed during the slow mixing (“RPM” (revolution per minute), *X*_3_). The range of the variables was determined following the sensitivity test and some additional preliminary experiments (data not shown): 1 to 3 for *X*_1_, 20 to 180 min for *X*_2_ and 20 to 70 rpm for *X*_3_. In addition, a separate preliminary experiment was conducted to compare the rapid mixing duration of 1, 2, 3, 5, 10 and 30 min, and 10 min was concluded as the optimum time.

**Figure 3:**
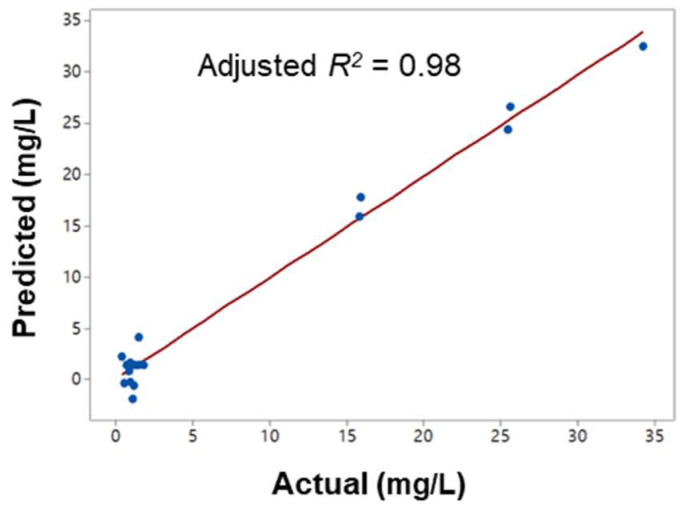
The actual and predicted [PO_4_-P] profiles after the coagulation process using PAC.

Eighteen conditions, including the quadruple trials at the center point, were assessed for their phosphate removal efficiencies (Table 2 and Table S1). While the RSM analysis was not able to produce a suitable model for FeCl_3_ due to lack-of-fit, the analysis has successfully derived a quadratic model to estimate the residual phosphate concentration for PAC (Equation (7)):

*Y_m_* = 58.26 − 55.34*X*_1_ + 0.1550*X*_2_ + 0.048*X*_3_ + 11.56*X*_1_^2^ − 0.000055X_2_^2^ − 0.00134*X*_3_^2^ − 0.04222*X*_1_*X*_2_ + 0.0445*X*_1_*X*_3_ − 0.000501*X*_2_*X*_3_ (7)

where *Y_m_* is the residual [PO_4_-P], *X*_1_ is the coagulant dosage, *X*_2_ is the slow mixing duration and *X*_3_ is the agitation speed during the slow mixing. This model fit well with the actual data (*R*^2^ = 0.916, *p* = 0.007, Table 4) with no significant lack-of-fit (*p* > 0.05) and a good agreement between the two datasets (Table 2, Figure 3). Thus, this model (Equation (7)) can be regarded as an adequate estimation of the responses of the independent variables to the dependent variable within the defined region.

**Figure 4:**
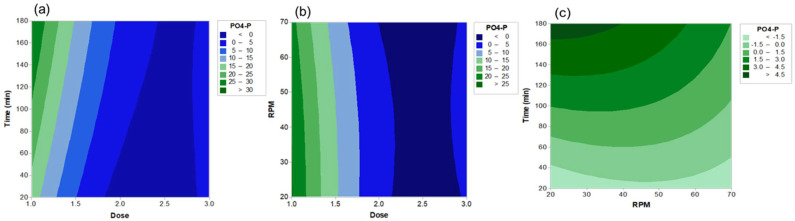
Contour plots of the responses of the residual [PO_4_-P] according to PAC dose (Dose), slow mixing time (Time) and slow mixing speed (RPM). Cross-sections are shown for (**a**) Dose × Time, (**b**) Dose × RPM or (**c**) Time × RPM.

The significance of the model terms was verified using ANOVA (Table 4). Out of the three, only one linear term, D (*X*_1_), could be regarded significant (*p* < 0.05). This result agrees with the contour patterns where Dose is the major driver of the residual [PO_4_-P] (Figure 4a,b). Conversely, the slope of the contour according to Time (*X*_2_) and RPM (*X*_3_) variations was only mild (Figure 4c). Except for Dose × Dose, the quadratic and the interaction terms were not statistically significant (*p* > 0.05), and no clear pattern of interaction was observable from the RSM plots (Figure 4).

Overall, Dose was the sole parameter that significantly shaped the phosphate removal efficiency from the anaerobic sludge using PAC. This is in accordance with previous studies where the amount of added coagulant was considered as an important factor [10,17]. As the coagulation performance tends to saturate when a coagulant dose increases [18], optimization is crucial for the economic feasibility of the process. Depending on the coagulant type and target [PO_4_-P], a dose of 2–5 can be suggested from the results of this study. Because the effects of the slow mixing regime (i.e., the duration and the agitation speed) were unclear in this study, optimizing the rapid mixing conditions and minimizing the slow mixing step could be tested in future studies. However, as shown in the case of PAC + FeCl_3_ (Table 3), slow mixing conditions could affect the coagulation performance of a combinatory chemical. The effects of pH on the coagulation between PAC and phosphorus have been shown two-fold: improved coagulation at acidic conditions (3.0–5.5), on the one hand, and elevated Al(OH)_3(s)_ level at neutral range on the other hand [19]. Thus, it could be inferred that the neutral pH (~7.0) applied in the coagulation process in this study may have facilitated the coagulation process. One of the limitations of this study is that only one type of sludge (from a domestic wastewater treatment plant) was tested. Indeed, our preliminary experiments with two other sludge types (from a food waste digestion plant and a combined food waste and sewage sludge digestion plant) showed comparable phosphate removal performance (data not shown); a future study for various sludge types would be valuable. Finally, it should be noted that the [PO_4_-P] in the anaerobic sludge is generally higher than the total phosphorus levels in typical sewage (<10 mg/L) [20]; therefore, a further optimization study would be necessary for sewage [21].

## 4. Conclusions

The phosphate removal from AD sludge using coagulation was assessed in two steps. The sensitivity test compared five coagulant types to conclude that PAC (or combined PAC and FeCl_3_) would be the most efficient coagulant. The optimization study produced a suitable quadratic model for PAC but not for FeCl_3_. The dose of PAC was the significant variable for phosphate removal efficiency, while the effects of the duration and agitation speed for the slow mixing were limited. Following the results of this study, some future study directions could be suggested: (1) using different anaerobic sludge types, (2) optimizing rapid mixing conditions and (3) exploring different variable ranges for other coagulant types.

## Figures and Tables

**Table 1:** Characteristics of the anaerobic sludge samples (*n* = 4).

Parameter	Average	Standard Deviation	Minimum	Maximum
pH	7.5	0.2	7.2	7.7
Conductivity (μS/cm)	6.0	0.7	5.2	6.9
Total solids (mg/L)	21,855	2252	20,360	25,085
Volatile solids (mg/L)	15,281	1576	14,115	17,535
Total phosphorus (mg/L)	989	186	789	1197
PO_4_^3—^P (mg/L)	169.2	10.8	153.4	177.9
Total nitrogen (mg/L)	2388	414	1963	2745
NH_4_^+^-N (mg/L)	634	108	480	730
Mg (mg/L)	7.1	0.4	6.6	7.6

**Table 2:** The experimental design and results for the optimization study using PAC.

Run Number (Randomized)	CCD Condition			Residual [PO_4_−P] (mg/L)	
	Dose (*X*_1_)	Time (*X*_2_) (min)	RPM (*X*_3_) (rpm)	Actual	Predicted
1	2	100	45	1.500	1.538
2	1	180	70	25.650	26.632
3	3	180	70	1.180	−0.554
4	3	20	70	0.370	2.271
5	2	100	45	1.275	1.583
6	1	20	70	15.800	15.948
7	3	20	20	0.520	−0.286
8	2	100	45	1.775	1.538
9	3	180	20	0.865	0.894
10	1	180	20	34.250	32.525
11	2	100	45	1.250	1.538
12	1	20	20	15.925	17.836
13	1	100	45	25.475	24.424
14	2	180	45	1.440	4.153
15	2	100	70	0.900	−0.132
16	3	100	45	0.895	1.770
17	2	20	45	1.110	−1.779
18	2	100	20	0.680	1.536

**Table 3:** The exponential decay constants (*b*) and the coefficients of determination (*R*^2^) derived from the sensitivity test.

Coagulant	50 rpm		20 rpm	
	*b*	*R* ^2^	*b*	*R* ^2^
Alum	0.687	0.991	0.679	0.994
PAC	1.650	0.999	1.522	0.999
FeCl_2_	0.805	0.993	0.825	0.999
FeCl_3_	0.765	0.994	0.621	0.997
PAC + FeCl_3_	3.639 (1.819) *	0.999	2.346 (1.173) *	0.999

* The decay constant was halved because the total dosage was double for PAC + FeCl_3_.

**Table 4:** The ANOVA results of the quadratic model for PAC derived from RSM.

Term	Degree of Freedom	*F*-Value	*p*-Value
Model	10	7.60	0.007
Dose (*X*_1_)	1	9.81	0.017
Time (*X*_2_)	1	3.71	0.095
RPM (*X*_3_)	1	0.72	0.426
Dose × Dose (*X*_1_^2^)	1	8.90	0.020
Time × Time (*X*_2_^2^)	1	0.25	0.632
RPM × RPM (*X*_3_^2^)	1	0.25	0.630
Dose × Time (*X*_1_ × *X*_2_)	1	2.54	0.155
Dose × RPM (*X*_1_ × *X*_3_)	1	4.50	0.072
Time × RPM (*X*_2_ × *X*_3_)	1	4.66	0.068
Lack-of-fit	4	4.62	0.120

## Supplementary Materials

The following supporting information can be downloaded at: https://www.mdpi.com/article/10.3390/ijerph19031693/s1, Table S1: The experimental design and results for the optimization study using FeCl_3_.
