# Supplementary material for: Responses of Coagulant Type, Dosage and Process Conditions to Phosphate Removal Efficiency from Anaerobic Sludge
Source: Int J Environ Res Public Health. 2022 Feb 1;19(3):1693. doi: 10.3390/ijerph19031693 (PMC8835144; doi:10.3390/ijerph19031693)
Supplement: Supplementary file 1 [file ijerph-19-01693-s001.zip › ijerph-1570901-SI.pdf]

International Journal of
Environmental Research and Public Health

MDPI

Table S1. The experimental design and results for the optimization study using $FeCl_3$. No significant model (linear nor quadratic) was derived due to lack-of-fit.

Run number (randomized)	CCD* condition			Residual [PO_4-P] (mg/L)
	Dose (X_1)	Time (X_2) (min)	RPM** (X_3) (rpm)	
**1**	2	100	70	15.440
2	3	100	45	2.970
3	2	180	45	17.670
4	2	20	45	15.700
5	1	100	45	99.150
6	2	100	20	9.190
7	1	180	20	38.925
8	1	20	70	44.325
9	2	100	45	13.225
10	1	180	70	52.100
11	2	100	45	14.100
12	3	180	20	1.005
13	2	100	45	14.300
14	3	180	70	2.420
15	2	100	45	15.525
16	3	20	70	0.805
17	3	20	20	0.715
18	1	20	20	36.575

* Central composite design. ** Revolution per minute (slow mixing agitation speed).
